# A pragmatic controlled trial to prevent childhood obesity within a risk group at maternity and child health-care clinics: results up to six years of age (the VACOPP study)

**DOI:** 10.1186/s12887-018-1065-3

**Published:** 2018-02-27

**Authors:** Taina Mustila, Jani Raitanen, Päivi Keskinen, Riitta Luoto

**Affiliations:** 1 Seinäjoki Central Hospital, Hanneksenrinne 7, 60220 Seinäjoki, Finland; 2 UKK Institute for Health Promotion, Tampere, Finland; 3 Faculty of Social Sciences, University of Tampere, Tampere, Finland; 4 Pediatric Research Centre, 33014 University of Tampere, Tampere, Finland; 5 Tampere University Hospital, 33521 Tampere, Finland

**Keywords:** Childhood obesity, Prevention, Diet, Physical activity, Intervention, Gestational diabetes mellitus, Pragmatic

## Abstract

**Background:**

Obesity in childhood appears often during the toddler years. The prenatal environment influences obesity risk. Maternal gestational diabetes, the child’s diet, and physical activity in the first few years have an important role in subsequent weight gain. A study was conducted to evaluate effectiveness of a primary health-care lifestyle counselling intervention in prevention of childhood obesity up to 6 years of age.

**Methods:**

The study was a controlled pragmatic trial to prevent childhood obesity and was implemented at maternity and child health-care clinics. The participants (*n* = 185) were mothers at risk of gestational diabetes mellitus with their offspring born between 2008 and 2010. The prenatal intervention, started at the end of the first trimester of pregnancy, consisted of counselling on diet and physical activity by municipal health-care staff. The intervention continued at yearly appointments with a public health-nurse at child health-care clinics. The paper reports the offspring weight gain results for 2–6 years of age. Weight gain up to 6 years of age was assessed as BMI standard deviation scores (SDS) via a mixed-effect linear regression model. The proportion of children at 6 years with overweight/obesity was assessed as weight-for-height percentage and ISO-BMI. Priority was not given to power calculations, because of the study’s pragmatic nature.

**Results:**

One hundred forty seven children’s (control *n* = 76/85% and intervention *n* = 71/56%) weight and height scores were available for analysis at 6 years of age. There was no significant difference in weight gain or overweight/obesity proportions between the groups at 6 years of age, but the proportion of children with obesity in both groups was high (assessed as ISO-BMI 9.9% and 11.8%) relative to prevalence in this age group in Finland.

**Conclusion:**

As the authors previously reported, the intervention-group mothers had lower prevalence of gestational diabetes mellitus, but a decrease in obesity incidence before school age among their offspring was not found. The authors believe that an effective intervention should start before conception, continuing during pregnancy and the postpartum period through the developmentally unique child’s first years.

**Trial registration:**

ClinicalTrials.gov NCT00970710. Registered 1 September 2009. Retrospectively registered.

## Background

The prevalence of obesity even among pre-school-age children has increased in recent decades, and this is a global trend [1]. Overweight and obesity prevalence is significant already in the pre-school years: 16.1% and 3.9% of five-year-old girls and 7.5%/3.0% of boys of the same age in Finland are reported to have overweight and obesity, respectively [2]. Early adiposity rebound (AR) has been found to be a marker of higher risk for obesity in children and youth; AR is the point of minimal body mass index (BMI) before the second rise in the BMI curve in childhood, normally between five and 7 years of age (AR is considered to be early if it occurs before the age of 5 years) [3, 4]. Pre-schoolers with obesity tend to become schoolchildren and teenagers with obesity, which leads to increased risk of cardiovascular disease in adulthood and to an intergenerational cycle of these health problems [5, 6]. Results of obesity treatment are not encouraging, and prevention of excess weight gain is considered the most effective way to reduce obesity prevalence both during childhood and in adulthood. Early-childhood obesity has a multifactor origin [7, 8]. Prenatal modifiable factors suspected to promote obesity are mother’s obesity before pregnancy, gestational diabetes mellitus (GDM), and smoking during pregnancy, with another being excessive weight gain during pregnancy [7, 9–14], and GDM appears to increase the risk of obesity in offspring even in cases of normal birth weight [15–17]. Large-for-gestational-age newborns have been shown to have a higher risk of obesity; also, infant feeding, sleep duration, and rapid weight gain in the first few months have been shown to influence the risk of children gaining excess weight [18–27].

In light of these potential risk factors, obesity prevention should start early in life. The widespread problem of obesity calls for preventive means that can be integrated into existing health-care settings and also for changes in society that contribute to healthy weight gain in the population [7, 8]. Pragmatic trials are aimed at finding effective preventive programmes that could be incorporated into the usual health-care system [28]. Pregnant mothers and families with small children visit child-welfare clinics regularly in primary health care. They are also interested in the wellbeing of their offspring and hence are receptive to lifestyle counselling. Dietary and physical-activity habits are modifiable during the pre-school years [29, 30]. With lifestyle counselling, a significant effect can be achieved when the target group are known to be at risk of gaining excessive weight. Mothers at risk of developing GDM and their offspring are one such risk group [7]. This group includes pregnant mothers with overweight or obesity, mothers with a history of GDM, a macrosomic newborn or close relatives with type 2 diabetes [31]. These mothers may also have a hereditary predisposition to obesity and type 2 diabetes, with a high risk of passing these risks to their offspring.

To the best of our knowledge, no previous results have been published from intervention studies that have aimed primarily at the prevention of obesity among the offspring and that have started during or before the first trimester of pregnancy. This is at odds with the growing evidence that the time before conception, the prenatal and perinatal periods, and early childhood are the critical windows for effective prevention. Some obesity-prevention studies targeted at infancy have been reported on, with most involving short intervention and follow-up periods [32, 33]. The effect on children’s adiposity or weight development, if any, has been found to be slightly positive [34–36]. Some randomised studies are still in progress [37–39]. There are a few studies, originally examining pregnancy outcomes such as excess weight gain during pregnancy, prevention of GDM, or postpartum weight retention, in which, additionally, the offspring’s weight development was evaluated for 1–7 years of age [40–44]. Intensified counselling on diet and physical activity (PA) directed at mothers during the infant’s first year resulted in offspring’s slower weight gain by the age of 4 years in a cluster-randomised pilot study [45]. This intervention when applied during pregnancy did not have the same effect on offspring weight [41]. In a study by Gillman et al., treatment of mild GDM had no effect on the offspring’s weight gain by age 4–5 years [40], and likewise no effect on pre-school weight gain was found for the gestational lifestyle intervention of the NELLI Study, the Lifestyle in Pregnancy and Offspring (LiPO) study, or the study by Vesco et al. of a weight-management intervention for limiting gestational weight gain (GWG) a in a group of women with obesity [42–44]. Evidence from the obesity-prevention programmes reported upon has shown that multifaceted intervention could be more effective than targeting a single behaviour [7, 32].

The main results of the controlled lifestyle intervention designed to prevent obesity before school age (the VACOPP, or Vaasa Childhood Obesity Primary Prevention, study) are reported here [46]. The setting of the study was maternity and child health-care clinics in the city of Vaasa, in Western Finland. The intervention started at the end of the first trimester for pregnant mothers and continued with their families until the child was 5 years old. The outcomes presented here cover the offspring’s weight gain along with overweight and obesity incidence in the trial groups until the age of 6 years.

## Methods

### Design and participants

Our study was a non-randomised pragmatic controlled clinical trial. All maternity and child health-care clinics in the city of Vaasa, in Western Finland, participated in the recruitment and intervention. The subjects were recruited from among all eligible GDM risk-group mothers in this city during the chosen recruitment period. Each municipal maternity and child health-care clinic in the city participated in the recruitment. A study nurse recruited GDM risk-group mothers and their offspring born in 2008 to the control group before the offspring reached 1 year of age. The intervention-group mothers were recruited from among the GDM risk-group mothers who were pregnant between February 2009 and April 2010 by public-health nurses. Their offspring comprise the intervention-group children. These criteria were applied for GDM risk: body mass index (BMI) ≥ 25 kg/m^2^, macrosomic newborn (birth weight ≥ 4500 g), GDM in any previous pregnancy or an immediate family history of diabetes, and/or age ≥ 40 years. The exclusion criteria were having a multiple pregnancy, being unable to speak Finnish, engaging in substance abuse, and displaying severe psychiatric problems.

Our study was a pragmatic trial, which is why we decided not to give priority to power calculations. In the city chosen, relatively limited number of mothers were expected to participate in the study, so statistical significance in a rigorous sense could not be demanded. The estimate of the mean BMI *z*-score for the control-group offspring is a rough one and yields only an inaccurate power calculation [47]. The design and participants were described in more detail in the protocol article [46].

### Intervention

The two group counselling sessions were held in the first and the second trimester of pregnancy. A physiotherapist and a dietician in public health care were the teachers. The recommended consumption of fibre, energy content, quality of carbohydrates, and fat in the diet were emphasised [48]. Mothers were advised to exercise for at least 2.5 h/week (until at least slightly out of breath) and to engage in muscle training twice a week, taking into account what is suitable exercise for pregnant women [49]. The mothers were told also that a healthful diet, exercise, and appropriate weight gain during pregnancy help to prevent GDM, act against perinatal problems for the newborn, and favour the child’s healthy weight gain. During the 13 routine visits to the maternity health-care clinics, starting with the tenth week of pregnancy, the public-health nurse (PHN) briefly repeated the counselling to the mother. Breastfeeding until the child is 6 months old was recommended. Intervention-group children had a 30–60-min longer appointment with a PHN at the child health-care clinic at the routine yearly control visits for 1–5 years of age. Counselling on diet, age-appropriate physical exercise, sleep, and screen time was given. The counselling employed a motivating interview method endorsed by the Finnish Heart Association, called ‘Smart Family’ [46]. The intervention has been described in more detail in the protocol article [46].

### Outcome measures

The primary outcomes were BMI-SDS development until age 6 years and the proportion of children at the age of 6 years with overweight or obesity as measured via weight-for-height percentage and ISO-BMI. Weight-for-height curves with percentage deviation of the mean for evaluating overweight/obesity in children are preferred in Finnish health care in addition to ISO-BMI, which is the BMI level equivalent for overweight and obesity in adulthood (≥ 25 kg/m^2^ and ≥30 kg/m^2^, respectively) were the child’s BMI to stay the same until adulthood. The new Finnish growth reference was used [50]. Pregnancy, newborn, and infant outcomes have already been reported [51]. The parents’ education levels are defined thus: ‘low’ corresponds to education as far as vocational school; ‘medium’ indicates a polytechnic degree and ‘high’ a university degree (Table 1). The secondary outcomes have been described in the protocol article and in a previous report on this study [46].

**Table 1:** Baseline characteristics of the trial groups participating in the study at offspring age of six years (mean or frequency and 95% confidence interval^*^)

	Intervention	Control	*p*-value	Missing
*N*	71	76		
Age of mother before pregnancy (years)	31.8 (30.4 to 33.1)	30.2 (29.0 to 31.5)	0.09^a^	–
Mother’s education			0.90^c^	–
Low	29.6% (20.2% to 41.0%)	27.6% (18.8% to 38.6%)		
Medium	42.2% (31.5% to 53.8%)	46.1% (35.3% to 57.2%)		
High	28.2% (19.0% to 39.5%)	26.3% (17.7% to 37.2%)		
Father’s education			0.30^c^	–
Low	31.0% (21.4% to 42.5%)	35.5% (25.7% to 46.7%)		
Medium	35.2% (25.1% to 46.8%)	42.1% (31.6% to 53.3%)		
High	33.8% (23.9% to 45.4%)	22.4% (14.5% to 32.9%)		
Mother’s pre-pregnancy BMI (kg/m^2^)	27.4 (26.3 to 28.5)	26.6 (25.6 to 27.5)	0.25^a^	–
Proportion of mothers with obesity (BMI ≥ 30 kg/m^2^)	23.9% (15.5% to 35.0%)	18.4% (11.3% to 28.6%)	0.41^c^	–
Father’s BMI (kg/m^2^)	26.9 (26.0 to 27.7)	27.1 (26.1 to 28.1)	0.69^b^	2, 3
Proportion of fathers with obesity (BMI ≥ 30 kg/m^2^)	17.4% (10.2% to 28.0%)	16.4% (9.7% to 26.6%)	0.88^c^	2, 3
Mother, type 2 diabetes	0.0% (0% to 5.2%)	1.3% (0.2% to 7.1%)	1.00^d^	1, 0
Father, type 2 diabetes	1.4% (0.3% to 7.8%)	1.4% (0.2% to 7.3%)	1.00^d^	2, 2
Proportion of grandparent with obesity (BMI ≥ 30 kg/m^2^)	52.3% (40.4% to 64.0%)	56.3% (44.8% to 67.3%)	0.38^c^	6, 5
Parity			0.28^c^	–
Primiparous	59.2% (47.5% to 69.8%)	46.0% (35.3% to 57.2%)		
Second pregnancy	23.9% (15.5% to 35.0%)	32.9% (23.4% to 44.1%)		
At least third pregnancy	16.9% (9.9% to 27.3%)	21.1% (13.4% to 31.5%)		
History of newborn > 4500 g	2.9% (0.8% to 9.8%)	3.9% (1.4% to 11.0%)	0.72^c^	1, 0
Mother smoking during pregnancy	1.4% (0.2% to 7.6%)	9.2% (4.5% to 17.8%)	0.04^c^	–
Mother’s physical activity (hours/week) during first trimester of pregnancy (before intervention)	4.5 (3.7 to 5.2)	4.5 (3.6 to 5.3)	0.38^b^	2, 3
OGTT (weeks 26–28 of gestation)				
Pathological OGTT result (0 h ≥ 5.3 or				
1 h ≥ 10.0 or 2 h ≥ 8.6 mmol/l)	23.9% (15.5% to 35.0%)	46.1% (35.3% to 57.2%)	0.01^c^	–
Gestational weight gain until 37 weeks (kg)	11.3 (10.2 to 12.4)	12.9 (11.6 to 14.3)	0.08^a^	2, 0
Neonatal outcomes				
Gestational age at birth	39.5 (39.1 to 39.9)	39.4 (39.1 to 39.7)	0.53^b^	–
Sex of newborn (boy)	53.5% (42.0% to 64.6%)	52.6% (41.6% to 63.5%)	0.91^c^	–
Birth weight (grams)	3455 (3333 to 3576)	3509 (3407 to 3611)	0.49^a^	–
Large for gestational age	5.6% (2.2% to 13.6%)	6.6% (2.8% to 14.5%)	0.81^c^	–
Exclusive breastfeeding (months)	3.2 (2.7 to 3.7)	2.7 (2.2 to 3.2)	0.16^b^	1, 0

^a^Independent-samples *t*-test. ^b^Mann–Whitney U-test. ^c^Chi-squared test. ^d^Fisher’s exact test

*Wilson score method for interval without continuity correction

*BMI* body mass index, *OGTT* oral glucose tolerance test (2-h)

### Data collection

Child’s weight was measured to the nearest 0.1 kg with the child in light clothing on a standard electronic scale by child health-care clinic PHNs at yearly appointments near the child’s birthday. Height too was measured during these visits, to the nearest 0.1 cm with a standard stadiometer. The study questionnaires were completed by the parents at these appointments or shortly thereafter. The PHNs submitted the completed questionnaires, along with the child’s weight, height, blood pressure, and waist circumference measures. These measurements were recorded also in the health-care centre’s electronic database, from which the researcher could check them if needed. Long-term illnesses affecting growth (e.g., severe food allergies) were recorded via this questionnaire. The content of the questionnaire form in full and a description of all data items collected were reported upon in the study protocol article [46].

### Statistical methods

The characteristics of the study participants are described in terms of means or frequencies and 95% confidence intervals (Tables 1, 2 and 3). The 95% confidence intervals (CIs) were calculated for continuous variables via the formula mean ± (1.96 * standard error of the mean) and for categorical variables via the Wilson score method without continuity correction, in accordance with Newcombe’s work [52]. Group differences were evaluated via Student’s *t*-test or Mann–Whitney U-test for normally or non-normally distributed continuous variables. Normality was assessed through examination of the skewness and kurtosis of the distributions. Categorical variables were tested via chi-squared test or Fisher’s exact test.

**Table 2:** Estimates and 95% confidence intervals for BMI-SDS from two to six years – results from a multilevel mixed-effects linear regression model including group (*n* = 171), age and sex of the child; pre-pregnancy BMI of the mother, and interaction between group and age of the child

	Coefficient (95% CI)	*p*-value
Group (ref. = control)	−0.02 (− 0.70 to 0.65)	0.94
Age of the child	−0.23 (− 0.44 to 0.02)	0.03
Age of the child^2^	0.03 (0.00 to 0.05)	0.04
Group ^*^ Age of the child	0.02 (−0.28 to 0.32)	0.89
Group * Age of the child^2^	−0.00 (− 0.04 to 0.03)	0.81
Maternal pre-pregnancy BMI	0.01 (0.00 to 0.03)	0.02
Sex of the child	−0.02 (− 0.32 to 0.28)	0.88
Constant	−0.41 (−1.38 to 0.55)	0.40

*BMI* body mass index, *SDS* standard deviation score

**Table 3:** Proportions of children in the study groups at 6 years of age with overweight or obesity (proportion and 95% confidence interval) assessed as ISO-BMI or weight-for-height percentage, where adiposity rebound is presented in two classes

	Intervention	Control	*p*-value	Missing
*N*	71	76		
Overweight at six years of age				
ISO-BMI ≥ 25	18.3% (11.0% to 28.8%)	19.7% (12.3% to 30.0%)	0.83^a^	
Weight for height ≥ + 10%	20.0% (12.3% to 30.8%)	22.4% (14.5% to 32.9%)	0.73^a^	
Obesity at six years of age				
ISO-BMI ≥ 30	9.9% (4.9% to 19.0%)	11.8% (6.4% to 21.0%)	0.70^a^	
Weight for height > + 20%	12.9% (6.9% to 22.7%)	13.2% (7.3% to 22.6%)	0.96^a^	
Adiposity rebound			0.69^a^	2, 1
Early (< 5 years)	29 (42.0%)	34 (45.3%)		
Normal (≥ 5 years)	40 (58.0%)	41 (54.7%)		

^a^Chi-squared test

ISO-BMI, BMI level equivalent for overweight and obesity in adulthood

The difference in the development of child weight gain between the groups (intervention vs. control) was analysed as BMI-SDS by means of a multilevel mixed-effect linear regression model so as to take into account the within-child correlation between repeated measures. This model included a variable (group) to indicate the difference between groups at baseline and another (age of child) to indicate the changes in BMI-SDS over time. The difference in the change in BMI-SDS from two to 6 years of age between the two groups was tested with a term for interaction between group and age of child. To allow for a non-linear individual-specific trajectory across time, a quadratic term for age was included. In addition, we added potential confounding variables to the model: mother’s pre-pregnancy BMI and gender of the child. Since BMI-SDS can be calculated from 2 years of age [50], this analysis included 171 children. Overweight or obesity was assessed in terms of weight and height converted to weight-for-height percentages and also ISO-BMI (again, the BMI level equivalent for adulthood overweight and obesity (≥ 25 kg/m^2^ and ≥30 kg/m^2^, respectively) if the child’s BMI level were to stay the same until adulthood) in accordance with the Finnish growth reference [50]. In this study, AR was considered to be early if the child’s BMI was lowest at two, three, or 4 years of age and normal if it was lowest at age five or 6 years in this group of 2–6-year-old children. All analyses were performed by means of Stata software (version 13.1 for Windows), from StataCorp LP, Texas, USA.

## Results

**Fig. 1:**
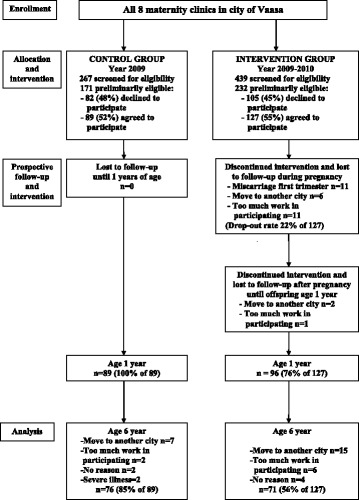
Flowchart

The study flow is described in Fig. 1. Roughly 700 women per year give birth in the city of Vaasa. In the intervention group, the offspring of 71 of the 127 mothers who started the intervention in pregnancy (56%) were still taking part in the study when the child was 6 years old (i.e., at the planned end of the study), with the corresponding figure for the control group being 76 out of 89 children (85%). Most of the dropouts were cases of moving to another city and hence being unable to remain in the study. We analysed baseline characteristics that might interfere with offspring weight development with regard to those children whose anthropometrics were available when they were 6 years old (*n* = 147) and found no statistically significant differences between the groups (Table 1). The baseline characteristics of children whose anthropometrics were available at age 1 year (*n* = 185) have already been reported [51].

**Fig. 2:**
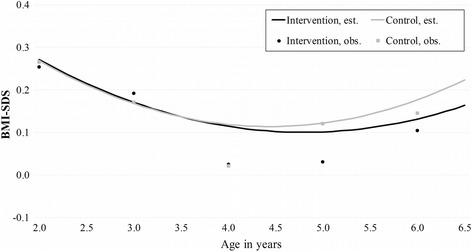
BMI-SDS in whole group (*N* = 171) from two years to six years of age. Non-linear model including age of child, mother’s pre-pregnancy weight, and group × age interaction. Obs., observed; Est., estimated

According to the linear mixed-effects model, the BMI-SDS slopes did not differ significantly between the intervention and the control group (the *p*-values for linear and quadratic interactions were 0.89 and 0.81) (Table 2, Fig. 2). Adding gender and mother’s pre-pregnancy BMI to the model did not fundamentally affect the results. The proportions (expressed as percentage value deviation from the mean weight-for-height value in line with the Finnish definition of pre-school-age overweight and obesity) of children at the age of 6 years with at least overweight (≥ + 10% weight for height) or with obesity (≥ + 20% weight for height) were not significantly different between the groups. The result was the same when at least overweight and obesity were assessed as ISO-BMI (≥ 25 kg/m^2^ and ≥30 kg/m^2^, respectively) (Table 3). The difference in equivalent proportions of early adiposity rebound (< 5 years) between the two groups was not significant either (controls 34/45.3% vs. intervention 29/42.0%, *p* = 0.69) (Table 3).

## Discussion

The main result found for our pragmatic lifestyle intervention was a lower occurrence of GDM in the intervention group than in the control group, which result was reported earlier [51], [Table 1]. However, whether the intervention was effective in decreasing excessive weight gain among offspring remains an open question. The non-significant finding might be due also to the low power of the study causing failure to reveal differences between the groups. It has been shown that lower gestational glucose levels may be correlated with a child’s lower obesity and type 2 diabetes risk [11, 12, 17]. Rapid weight gain during the first year of life has been demonstrated to predict risk for later obesity [22]. In our study, the offspring’s weight gain up to 12 months of age did not differ significantly between groups, but there were slightly more children with overweight in the control group by 1 year of age [51]. Likewise, rapid weight gain in subsequent pre-school years seems to predict obesity in the school years [23]. In addition, early adiposity rebound has been shown to precede obesity in childhood and adulthood and to be a marker of cardiometabolic risk [3, 53]. In our study, no significant difference in the groups’ proportions of early vs. normal AR was found, but the proportion of children with early AR in both groups was high, predicting the offspring having the same metabolic risk as their mothers. The proportion of children at the age of 6 years with obesity in both group was high as well (defined as weight for height 12.9% and 13.2%) [2]. These results confirm that our target group for such an intervention may be appropriately chosen.

The offspring’s BMI was analysed and adjusted in accordance with the Finnish growth reference, for obtaining the SDS [50]. Weight gain was assessed with a linear mixed-effects model, which allows for a difference between the groups at baseline, intervention effects, and changes over time. No significant differences between the intervention and control group’s offspring weight gain during the first year or up to 6 years of age were found. Given that improvements in foetal conditions – such as the mother having a better glucose balance during pregnancy – have been shown to correlate with a good effect on offspring weight gain that emerges in the toddler years. Based on this our intervention had potential to diminish children’s overweight/obesity prevalence by age six [11, 12, 17]. However, as we have noted, the insufficient power of the study may have affected the results in this respect.

The overall dropout rate for the intervention group up to 6 years of age was 44% (Fig. 1). The most common reasons for dropping out were moving to a city out of reach of this intervention and the parents experiencing the study intervention or completing the questionnaires as too taxing. Furthermore, the recommendation to participate in blood tests every 2 years was felt to be too taxing for the child in many families, creating reluctance to take part in the study even despite the option of skipping the tests. The dropout rate in our study is acceptable in view of its longer-term intervention and follow-up. There were also dropouts in the control group (15% by age 6 years). It is possible that those families with the healthiest lifestyle and lowest risk of offspring’s excess weight gain were more likely to remain in the study, thereby diminishing the difference in proportions of children with overweight and obesity between groups. However, the baseline characteristics were comparable between groups at both 1 year and 6 years of age.

Our target group was mothers at risk of developing GDM and their offspring with a higher risk of unhealthy weight gain. The intervention extended across foetal, infant, and pre-school life, known times of risk for development of obesity. Almost 98% of the mothers in Finland visit municipal maternity health-care facilities, and the high participation percentage holds for child health-care clinic visits. If the intensified counselling is offered during these routine visits, the at-risk families are conveniently reached. However, those routine visits to child health-care clinics take place only once a year, which may entail too light an intervention for this risk group. Also, evidence is growing that intervention for this purpose should start even before pregnancy, to improve the mother’s metabolic health and hence a better prenatal environment in regard of the child receiving a healthier epigenetic heritage [54]. One marked problem is how to reach the risk group with childbearing potential for intensive counselling before pregnancy. Child health-care clinics may be a useful environment for targeting mothers with small children before pregnancy, but this is not true for first-time mothers. In addition, since obesity tends to begin in the early years, focusing more intensive lifestyle counselling also on offspring age 0–2 years within risk-showing families could be effective.

Our study had several limitations. It was not randomised, and the power may not have been sufficient to reveal statistically significant results. We believe also that, as the difference in study-group BMIs proved to be so small, precise primary power calculations would not have shown the number of participants to be sufficient for statistical significance in this intervention trial. An additional factor is that we wanted to perform the trial in this specific relatively large city in Finland, where the protocol is the same across all maternity health care, thereby primarily comparable in that regard. For this pragmatic trial, a randomised controlled design was not considered feasible, because the randomisation process would have been very likely to further reduce the rate of participation in the trial. A case-control study design is the choice in intervention studies when randomisation is not feasible and the study groups are matched as in our study (Table 1). The study design was discussed also in the protocol article [46]. The control group was prospective only from offspring age of 1 year, which may have caused some bias in the results; however, our choice may also have eliminated a possible Hawthorne effect on the control group during the intervention during pregnancy. As is the case with any pragmatic trial, the effectiveness of the counselling situation as a whole might have varied greatly. For example, the motivation of PHNs may vary, and the need for PHN deputies occasionally has an influence on counselling. The recruitment of the intervention group and the paperwork for the study were considered burdensome by some PHNs, mainly for reason of their busy work schedule. Allocating enough time for PHNs to manage the risk-group intervention appointments is crucial also.

One element of our study in its defence is its implementation in real-life practice, which demonstrates the counselling’s ability to be a sustainable part of municipal health care. Also, the maternity and child health-care clinics have a good opportunity to identify those at risk for childhood obesity at a stage in life when favourable lifestyle changes promote the offspring’s health most. Targeting the at-risk population in a setting that all families in this life situation visit eliminates the risk of stigmatisation. The costs of this study were quite moderate, and the results are generalisable to normal health care, because the study was realised as a part of usual practice at maternity and child health-care clinics.

## Conclusions

Obesity with its expensive health effects and economic disadvantages challenges us to initiate solid preventive actions. Primary health-care, maternity, and child health-care clinics reach the beginning of the next generation. Preventive pragmatic trials in real-life settings are needed if we are to target obesity risk groups extensively and economically. In our study, the previously reported improved glucose tolerance during pregnancy demonstrated potential to have a good effect also on offspring weight gain. However, this effect could not be seen in the study. The offspring in both groups showed a high occurrence of early adiposity rebound and high prevalence of obesity, confirming their risk-group status. The knowledge now available suggests that preventive lifestyle interventions should start even before conception, to be able to influence the foetal environment effectively, and also focus on the child’s first 2 years, to cover this time with its special risk for obesity development. In addition to applying the right timing, there may be a need for putting more effort and time into the intervention if it is to result in obesity prevention in children in pragmatic settings in health care.

## Abbreviations

AR: Adiposity rebound
BMI: Body mass index
CI: Confidence interval
GDM: Gestational diabetes mellitus
GWG: Gestational weight gain
PHN: Public-health nurse
SDS: Standard deviation score
